# The status of epidermal growth factor receptor in borderline ovarian tumours

**DOI:** 10.18632/oncotarget.7257

**Published:** 2016-02-08

**Authors:** Rania Showeil, Claudia Romano, Mikel Valganon, Maryou Lambros, Pritesh Trivedi, Susan Van Noorden, Ruethairat Sriraksa, Dalal El-Kaffash, Nour El-Etreby, Rachael Natrajan, Letizia Foroni, Richard Osborne, Mona El-Bahrawy

**Affiliations:** ^1^ Department of Histopathology, Imperial College London, London, United Kingdom; ^2^ Department of Clinical Pathology, Faculty of Medicine, Alexandria University, Alexandria, Egypt; ^3^ Imperial Molecular Pathology Laboratory, Imperial College London, London, United Kingdom; ^4^ Breakthrough Breast Cancer Research Centre, The Institute of Cancer Research, London, United Kingdom; ^5^ Epigenetics Group, International Agency for Research on Cancer, Lyon CEDEX 08, France; ^6^ Obstetrics and Gynaecology Department, Faculty of Medicine, Alexandria University, Alexandria, Egypt; ^7^ Dorset Cancer Centre, Poole Hospital, Dorset, United Kingdom; ^8^ Department of Pathology, Faculty of Medicine, Alexandria University, Alexandria, Egypt

**Keywords:** EGFR, borderline ovarian tumours

## Abstract

The majority of borderline ovarian tumours (BOTs) behave in a benign fashion, but some may show aggressive behavior. The reason behind this has not been elucidated. The epidermal growth factor receptor (EGFR) is known to contribute to cell survival signals as well as metastatic potential of some tumours. EGFR expression and gene status have not been thoroughly investigated in BOTs as it has in ovarian carcinomas. In this study we explore protein expression as well as gene mutations and amplifications of *EGFR* in BOTs in comparison to a subset of other epithelial ovarian tumours.

We studied 85 tumours, including 61 BOTs, 10 low grade serous carcinomas (LGSCs), 9 high grade serous carcinomas (HGSCs) and 5 benign epithelial tumours. EGFR protein expression was studied using immunohistochemistry. Mutations were investigated by Sanger sequencing exons 18-21 of the tyrosine kinase domain of *EGFR*. Cases with comparatively higher protein expression were examined for gene amplification by chromogenic in situ hybridization. We also studied the tumours for *KRAS* and *BRAF* mutations.

Immunohistochemistry results revealed both cytoplasmic and nuclear EGFR expression with variable degrees between tumours. The level of nuclear localization was relatively higher in BOTs and LGSCs as compared to HGSCs or benign tumours. The degree of nuclear expression of BOTs showed no significant difference from that in LGSCs (mean ranks 36.48, 33.05, respectively, p=0.625), but was significantly higher than in HGSCs (mean ranks: 38.88, 12.61 respectively, p< 0.001) and benign tumours (mean ranks: 35.18, 13.00 respectively, p= 0.010). Cytoplasmic expression level was higher in LGSCs. No *EGFR* gene mutations or amplification were identified, yet different polymorphisms were detected. Five different types of point mutations in the *KRAS* gene and the V600E *BRAF* mutation were detected exclusively in BOTs and LGSCs.

Our study reports for the first time nuclear localization of EGFR in BOTs. The nuclear localization similarities between BOTs and LGSCs and not HGSCs support the hypothesis suggesting evolution of LGSCs from BOTs. We also confirm that *EGFR* mutations and amplifications are not molecular events in the pathogenesis of BOTs.

## INTRODUCTION

Borderline ovarian tumours (BOTs) are a heterogeneous group of tumours, comprising up to 10% of ovarian epithelial neoplasms [1]. Serous and mucinous varieties constitute the majority of BOTs and unlike ovarian carcinoma, they occur mostly in women of reproductive age [2]. The majority of BOTs behave in a benign fashion, yet there remain a percentage of tumours that may recur or progress in a manner similar to malignant tumours [3]. To date there are no definite clinical or molecular markers that can help identify high risk BOTs that may behave in an aggressive fashion. Identification of risk factors for tumour progression is a challenge that can only be addressed effectively if the pathogenesis of these tumours is revealed. Mutations in *KRAS* and *BRAF* genes have been extensively studied in BOTs, but it is not known whether these mutations alone are sufficient to induce BOTs in vivo [4]. Epidermal growth factor receptor (EGFR) is a trans-membrane receptor with tyrosine kinase activity that is important in cell growth and proliferation [5]. It is the main regulator of downstream molecules in a number of pathways including the *RAS* and *RAF* genes [6]. EGFR plays a role in cell survival signals as well as in epithelial to mesenchymal transition of tumour cells, angiogenesis and subsequent metastasis [7]. *EGFR* amplification, mutations and overexpression have been extensively and variably reported in ovarian carcinoma [8]. Increased EGFR expression has been correlated with poorer patient outcomes in some studies [8, 9]. As a consequence, EGFR has been a subject of investigation for targeted therapies, with monoclonal antibodies and small-molecule tyrosine kinase inhibitors, exploring it as a potential therapeutic agent in ovarian carcinoma. Very few reports included BOTs and mostly investigated a small number of tumours or studied a single aspect (protein expression or mutations only).

In this work, we studied EGFR protein expression, gene mutations and gene copy number in a large series of BOTs, to investigate whether EGFR plays a role in the pathogenesis of these tumours. We also aimed to explore any possible relation between EGFR protein expression and / or gene status and the presence of *KRAS* and *BRAF* mutations.

## RESULTS

We studied formalin fixed paraffin embedded tissues (FFPE) from eighty five cases of ovarian tumours including: 61 BOTs (including 45 serous, 14 mucinous, 1 endometrioid and 1 seromucinous), 10 low-grade serous carcinomas (LGSCs), 9 high grade serous carcinomas (HGSCs) and 5 benign epithelial tumours (2 serous cystadenomas and 3 mucinous cystadenomas).

### EGFR expression by immunohistochemistry

**Figure 1:**
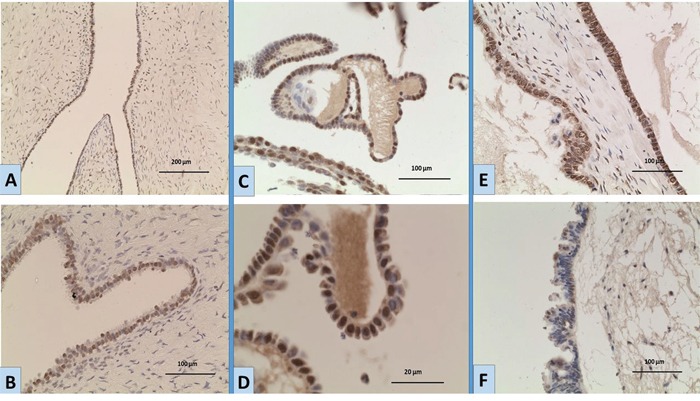
Expression of *EGFR* by immunohistochemistry in ovarian tumours **A, B.** a case of BOT showing moderate nuclear expression; [**A**: magnification x10; **B**: magnification; x20]. **C, D**. a BOT case showing nuclear expression of variable intensity [**C**: magnification x20; **D**: magnification x40]. **E.** a BOT case showing strong nuclear and moderate cytoplasmic expression [magnification x20]. **F.** a BOT case with negative nuclear staining and weak cytoplasmic expression [magnification x20].

Two distinct patterns of protein expression; nuclear and cytoplasmic, were detected in all tumour categories, Figure 1. No membranous staining was detected in any of the tumours. Using two different scoring systems (discussed in Methods), we compared the degree of nuclear and cytoplasmic expression between different tumour groups. Using the first scoring system (the H score [10], Table 1), we found that the level of nuclear expression in BOTs was significantly higher compared to HGSCs (mean ranks: 38.88, 12.61 respectively, p< 0.001) and compared to benign tumours (mean ranks: 35.18, 13.00 respectively, p= 0.010). On the other hand, no statistically significant difference existed between the level of nuclear positivity of BOTs and LGSCs (mean ranks 36.48, 33.05, respectively, p= 0.625). Combining BOTs and LGSCs as one category, their nuclear expression level came again higher in comparison to HGSCs (mean ranks: 43.74, 14.94, p< 0.001) and benign tumours (mean ranks: 40.11, 15.70, p= 0.017). This pattern of nuclear expression similarity between BOTs and LGSCs did not apply to cytoplasmic expression, where positive cytoplasmic expression in LGSCs came higher compared to all other tumour categories; BOTs (mean ranks: 52.00, 33.38, p= 0.008), HGSCs (mean ranks: 13.6, 6.0, p= 0.003), and benign tumours (mean ranks: 10.50, 3.00, p= 0.002).

**Table 1:** Mean ranks and p values obtained from comparing H- score [10] and Lo et al [11] score of EGFR expression in cases from two tumour categories (Group1 vs Group2)

Group 1	Group 2	H- score			Lo et al score		
		Mean rank (group 1)	Mean rank (group 2)	p value	Mean rank (group 1)	Mean rank (group 2)	p value
**Nuclear scoring results:**							
BOTs	LGSCs	36.48	33.05	p= 0.625	36.57	32.55	p= 0.567
BOTs	HGSCs	38.88	12.61	p< 0.001	38.97	12.06	p< 0.001
BOTs	Benign	35.18	13.00	p= 0.010	35.17	13.10	p= 0.013
BOTs and LGSC	HGSCs	43.74	14.94	p< 0.001	43.85	14.11	p< 0.001
BOTs and LGSCs	Benign	40.11	15.70	p= 0.017	40.11	15.17	p= 0.016
**Cytoplasmic scoring results:**							
BOTs	LGSCs	33.38	52.00	p= 0.008	34.20	47.00	p= 0.026
BOTs	HGSCs	36.21	30.67	p= 0.442	34.89	39.67	p= 0.437
BOTs	Benign	34.77	18.00	p= 0.059	34.52	21.10	p= 0.085
LGSCs	HGSCs	13.6	6.00	p= 0.003	11.00	8.89	p= 0.126
LGSCs	Benign	10.50	3.00	p= 0.002	9.50	5.00	p= 0.009

The Mann- Whitney's test is used in comparing the means of nuclear and cytoplasmic staining percentages between different groups. Significant differences are highlighted (p ≤ 0.05).

BOTs: borderline ovarian tumours, HGSCs: high grade serous carcinomas, LGSCs: low grade serous carcinomas

To further validate these results we employed a second scoring system that was used by Lo et al [11] (table 1, detailed in Methods); where only the percentage of positive cells is considered, regardless of the intensity of expression. This scoring system similarly showed no statistically significant difference between BOTs and LGSCs (mean ranks: 36.57, 32.55, p=0.567), while the there was a significant difference between BOTs and HGSCs (mean ranks: 38.96, 12.06, p< 0.001) and benign tumours (mean ranks: 35.17, 13.10, p= 0.013).

### *EGFR*, *BRAF* and *KRAS* gene mutation analysis

**Figure 2:**
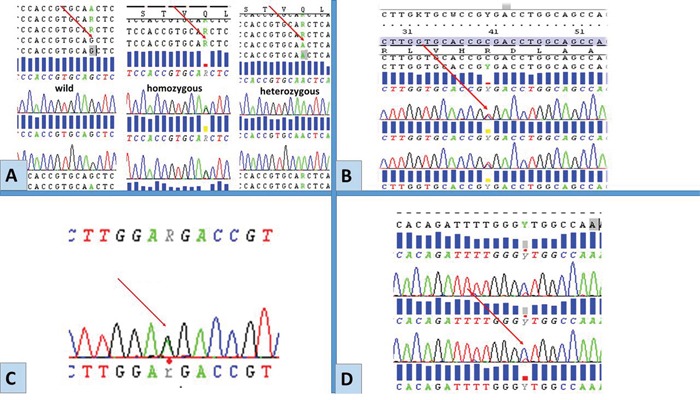
Representative chromatograms of mutations/SNPs revealed from sequences analysis of *EGFR* by Sanger sequencing Red arrows point to the site of mutations/SNPs: **A.** rs1050171, c.2361 G> A, exon 20. **B.** rs17290559, c.2508 C>T, exon 21. **C.** rs41420046, c.2487 G>, exon 21. **D.** COSM26129 c.2572 C>T (p.L858L), exon 21. These molecular variations were detected only in exons 20 and 21 among BOTs and LGSC.

No functional mutations (i.e. mutations altering gene product) in *EGFR* were detected in any of the tumours, yet sequence analysis revealed the presence of rs1050171, c.2361 G>A single nucleotide polymorphism (SNP) in exon 20. The genotypes GG (wild), GA (heterozygous) and AA (homozygous) were detected at different frequencies in different tumour groups Table 2 and Figure 2, 2A. In exon 21, three different single nucleotide substitutions were detected. These were the rs2229066, c.2508 C>T (in heterozygosity), detected in a total of 6 samples, a heterozygous rs41420046, c.2487 G>A in a serous BOT and the COSM26129, c.2572 C>T, L858L was identified in a different sample of serous BOTs (Figure 2, 2B-2D). Exons 18 and 19 were free from any molecular variation.

**Table 2:** Gene mutations and polymorphisms

		Borderline ovarian tumors n (%) ^(2)^	Low grade serous carcinoma n (%) ^(2)^	High grade serous carcinoma n (%) ^(2)^	Benign epithelial tumors n (%) ^(2)^
Gene Tumor					
**EGFR**^(1)^					
rs1050171 c.2361 G>A (exon 20)	Wild (G)	15 (24.6)	1 (10)	1 (11.1)	0 (0)
	Homozygous (A)	16 (26.2)	2 (20)	4 (44.4)	4 (80)
	Heterozygous (R)	30 (49.2)	7 (70)	4 (44.4)	1 (20)
rs2229066 c.2508 C>T (exon 21)		5 (8.2) (4 serous, 1 mucinous)	1 (10)	0 (0)	0 (0)
rs41420046 c.2487 G>A (exon 21)		1 (1.6) serous	0 (0)	0 (0)	0 (0)
COSM26129 c.2572 C>T, (exon 21) **L858L**		1 (1.6) serous	0 (0)	0 (0)	0 (0)
**KRAS**^(1)^					
c.34G>T **G12C**		3 (4.9) (2 serous, 1 mucinous)	1 (10)	0 (0)	0 (0)
c.35G>A **G12D**		12 (19.7) (8 serous, 4 mucinous)	4 (40)	0 (0)	0 (0)
c.35G>C **G12A**		1 (1.6) (0 serous, 1 mucinous)	0 (0)	0 (0)	0 (0)
c.35G>T **G12V**		6 (9.8) 3 serous, 2 mucinous, 1 seromucinous)	1 (10)	0 (0)	0 (0)
c.37G>A **G13S**		1 (1.6) (1 serous, 0 mucinous)	0 (0)	0 (0)	0 (0)
Total of KRAS mutations		23 (37.7)	6 (60)	0 (0)	0 (0)
**BRAF**^(1)^					
T>A		15 (24.6) 15 serous, 0 mucinous	4 (40)	0 (0)	0 (0)

(1) No concurrent mutations of both KRAS and BRAF existed in any case.

(2) Percentages do not add up to 100 due to cases without mutation/SNP

*KRAS* and *BRAF* mutations were detected exclusively in BOTs and LGSCs. For *KRAS*, five types of single substitutions in codons 12 and 13 were found, the most frequent was the G12D (19.7% in BOTs and 40% of LGSCs cases). The *BRAF* V600E mutation in exon 15 was detected in 24.6% of BOTs and 40% of LGSCs. No concurrent mutations of both *KRAS* and *BRAF* existed in any case. Table 2 shows the frequencies of all mutational analysis findings among the studied groups.

No significant association was found between the mean of nuclear expression of EGFR and the presence of mutation (p= 0.384), *BRAF* mutations (p= 0.553) or combined *KRAS* and *BRAF* (p= 0.598).

### *EGFR* gene amplification analysis by CISH

**Figure 3:**
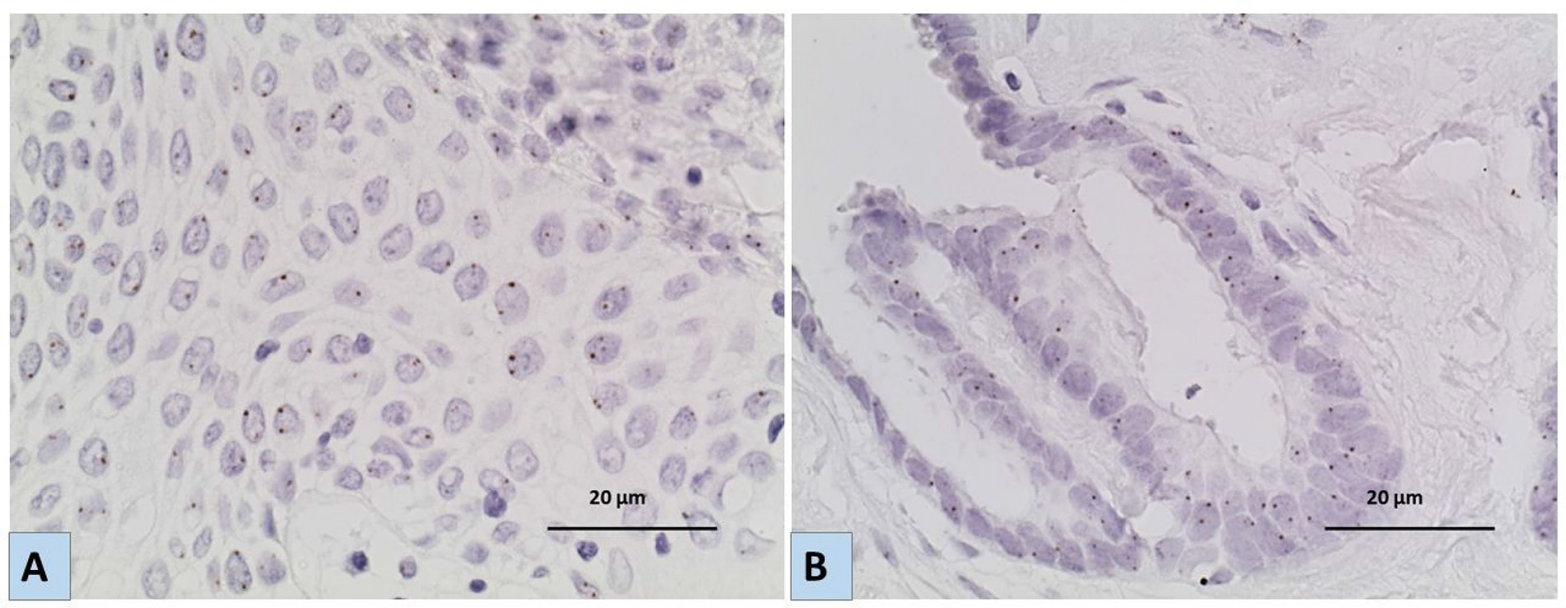
Representative images for the results of Chromogenic In situ hybridization (CISH) using *EGFR* specific probe **A.** a case of LGSC and **B.** a case of BOT showing no *EGFR* gene amplification/aneuploidy [magnification x40]. Six or more brown dots in the nucleus in more than 50% of tumour cells indicate amplification of the *EGFR* gene, while 3-5 are considered aneuploidy.

To ascertain whether high EGFR expression was related to *EGFR* gene amplification, we assessed amplification status in a subset (18 cases) of tumours that showed the highest expression using H score), which included 16 BOTs and 2 LGSCs. None of the cases showed gene amplification or aneuploidy. The range of *EGFR* gene copy number (GCN) in all cases was (1.16-2.30), median= 1.65, (Figure 3).

## DISCUSSION

The results of our study show the presence of nuclear localization of EGFR in ovarian tumours particularly in BOTs and LGSCs. The level of nuclear localization was higher in BOTs and LGSCs as compared to HGSCs or benign tumours. To our knowledge, this is the first report of nuclear expression of EGFR in BOTs, while it has been previously reported in some malignant tumour types including ovarian [12] and breast carcinoma [11, 13]. Xia et al conducted an immunohistochemical analysis for EGFR on 221 primary ovarian carcinomas of mixed grades, and he reported a 28.3% nuclear positivity among his cohort, and showed that nuclear expression correlated with low survival rates. Nuclear transport of EGFR family receptors has been previously reported, exerting several functions; mainly as a co- transcriptors and regulators of cyclin D1 promoter [14, 15].

In addition, different molecular and clinicopathologic studies suggest the evolution of LGSCs from BOTs, mostly through similarity in frequency of *KRAS* and *BRAF* mutations [4]. Our results showing significantly higher nuclear expression among these two categories compared to HGSCs and benign tumours, provide further support to this developmental relationship between serous BOTs and LGSCs, which is distinct from that of HGSCs [16, 17]. This also raises the possibility that EGFR may play a role in the pathogenesis of BOT-LGSC via nuclear localization, with no such role in the development of HGSCs.

Previous reports on the level of expression of membranous or cytoplasmic EGFR showed much variability, which may be due to differences in tissue preparation and fixation, different antibodies and staining protocols, but most importantly due to the great variability in scoring systems adopted. For our study we adopted the modified H- score method as it takes into consideration both percentages of cells showing expression as well as the intensity of the positive expression. We detected EGFR cytoplasmic expression in our cohort of tumours with variable distributions and intensity, where LGSCs showed the highest level of expression. In agreement with our results, Chen et al, showed that cytoplasmic EGFR positivity in both borderline and malignant ovarian tumours was significantly higher than in normal ovarian tissue and benign tumours [18]. Similarly, Nielsen et al reported that 67% of BOTs and 62% of ovarian carcinomas showed cytoplasmic positivity [19]. In contrast, Fujiwara et al, reported 0% positivity in his 10 studied BOTs, while 39.3% of the 152 serous carcinomas showed positive cytoplasmic staining [20]. Brustmann et al also reported no EGFR immune-reactivity in any of the serous cystadenomas or serous BOTs studied, compared to 64% of serous carcinomas [21]. In addition, van Haaften-Day compared the expression of EGFR in 16 invasive ovarian carcinomas and 11 serous borderline tumours and found that EGFR was expressed in a significantly greater fraction of malignant lesions (69%, n=16) than borderline lesions (18%, n=11) (p< 0.004) [22].

Our study shows that these patterns of EGFR expression are not related to underlying gene mutations or gene amplifications. In agreement with results of previous studies, our results confirm the rarity of *EGFR* mutations in BOTs [23, 24, 25]. However, we identified polymorphisms that have not been previously reported in ovarian tumours. Although being germline changes, some of these polymorphisms were previously reported to carry a potential relevance as prognostic markers. The identified rs1050171 polymorphism in exon 20 was previously reported to be associated with positive response to treatment with tyrosine kinase inhibitors (TKIs) in some cases of small cell carcinoma of the lung [26]. On the other hand, it was associated with worse survival rate in some oesophageal squamous cell carcinoma cases [27]. In addition, COSM26129 has only been described before in 3 cases of head and neck squamous cell carcinoma in 2007 [28]; our study is the second to report this variation and the first to report its presence in a case of serous BOT.

We detected *KRAS* and *BRAF* mutations exclusively in BOTs and LGSCs in our cohort and in frequencies comparable to previous reports for both *KRAS* (17-41%) and *BRAF* (30-71%) [6, 29–34]. Also in concordance with previous reports [32], *KRAS* and *BRAF* mutations were mutually exclusive in our study. We found no association between the level of expression of EGFR protein and *KRAS* and *BRAF* gene status. Szablewski et al [35] studied *KRAS* and *BRAF* mutations as well as EGFR expression in intestinal-type adenocarcinoma. Their results showed that *KRAS* mutations were frequent (42.9%) as was EGFR overexpression (63% of 43 samples), but similar to our findings, no statistically significant correlation between *KRAS* gene status and EGFR level of protein expression was detected.

Neither *EGFR* gene amplifications nor aneuploidy were detected in any of the BOTs or LGSCs we studied. Comparable results were reported by Dekanić et al, who reported a strong nuclear EGFR expression in colorectal carcinomas that was associated with cyclin-D1 but not with *EGFR* gene amplification [36]. But in contrast to these results, Lee et al found an increased copy number of the *EGFR* gene in 8.3% of BOTs and 37.8% of malignant tumours, but none in benign tumours [37].

The rarity of mutations and low frequency of *EGFR* polymorphisms detected and the lack of obvious amplification among our cohort suggests that these are not likely to be significant molecular events in the pathogenesis of BOTs or LGSCs. The high level of EGFR protein expression may be the result of other epigenetic or post transcriptional events rather than gene mutation or amplification.

## MATERIALS AND METHODS

### Selection of cases and ethical approvals

Tumour cases were retrieved from the Department of Histopathology, Hammersmith Hospital, London, UK. Slides from each case were reviewed and a representative block was selected with tumour content of at least 75%. The study was approved by The Hammersmith and Queen Charlotte's & Chelsea Research Ethics Committee.

### EGFR immunohistochemistry

Tissue sections of 5 μm thick were mounted on pre-coated slides, deparaffinised with xylene and rehydrated with graded ethanol. Endogenous peroxidase was blocked with Peroxidase Blocking Solution (DAKO, CA, USA) for 15 minutes. Sections were then incubated with primary antibody for EGFR (1:100, Clone: H11, Monoclonal Mouse Anti-Human, EGFR, Dako) [38] for overnight at 4°C. Positive controls were included for each run (Normal placental tissue) and a negative control slide was used for each case, as a duplicate section, where the antibody was replaced by antibody diluent. The reaction was visualised using Novolink™ Polymer Detection Systems (Leica Biosystems Newcastle Ltd, UK). Sections were counterstained with Haematoxylin. The stained slides were evaluated by two investigators (RS and ME) by light microscopy, using low (×10) and high (×20 or ×40) magnification. Discordant cases were jointly reviewed and a consensus was reached. The subcellular distribution of the staining between membrane, cytoplasm and nucleus was evaluated. For each case, reviewing the whole tumourous section, the percentage of positive cells and intensity of staining was estimated in each compartment. EGFR protein expression was scored in two different ways. First, using The H-score [10], which is the product of multiplication of the percentage of cells positive for membranous and cytoplasmic or positive for nuclear EGFR and the staining intensity, each producing a number (from 0 to 300), for both nuclear and cytoplasmic expression. Cytoplasmic or nuclear scoring was then graded as follows: grade 1 (score 0–100), grade 2 (101–200), and grade 3 (201–300). This was followed by calculating a combined score for cytoplasmic and nuclear together, ranging 2-6. The other scoring system considered only the percentage of positive cells regardless of the intensity, described by Lo et al [11], in which membranous or cytoplasmic EGFR staining was grouped into four groups: high (3, >50%), moderate (2, 26-50%), low (1, 1-25%), and negative (0, 0%), while nuclear EGFR was scored as high (3, >35%), moderate (2, 18-35%), low (1, 1-17%), and negative (0, 0%) as previously described.

### Mutation analysis using sanger sequencing

#### DNA extraction

Sections of 5-10 μm thickness were cut from FFPE tissue samples. Tumour tissue was scraped from at least 5 slides per case and genomic DNA was subjected to overnight digestion with sodium dodecyl sulphate and proteinase K (Sigma-Aldrich, USA) at 37°C, followed by standard phenol-chloroform (1: 1) extraction and ethanol precipitation. The precipitant was suspended in 35 μl of 10 mM Tris-Cl, of pH equals to 8.0. The quality of the genomic DNA was checked (OD A260/A280 > 1.7) spectrophotometrically, using NanoDrop 1000 Spectrophotometer V3.7, Thermo SCIENTIFIC.

### Polymerase chain reaction (PCR) for *EGFR*, *KRAS* and *BRAF*

Mutation analysis of exons 18-21 of tyrosine kinase domain of *EGFR*, exon 2 (codons 12 and 13) of *KRAS* and exon 15 of *BRAF* genes were performed by Polymerase chain reaction (PCR) amplification followed by direct sequencing of the PCR products. The primers used were provided from custom oligos, invitogen, life technologies ^TM^ and are listed in Table 3.

**Table 3:** Primers' sequences used for amplification of EGFR (exons 18-21), KRAS (exon 2) and BRAF (exon 15)

Gene	Forward primer (5′-3′)	Reverse primer (5′-3′)
**EGFR, exon 18**	TTGTCCTTCCAAATGAGCTG	ACAGCTTGCAAGGACTCTGG)
**EGFR, exon 19**	AGATCACTGGGCAGCATGT	CAGCTGCCAGACATGAGAAA
**EGFR, exon 20**	CATTCATGCGTCTTCACCTG	CATATCCCCATGGCAAACTC
**EGFR, exon 21**	ATCCTCCCCTGCATGTGTTA	CTCAGAGCCTGGCATGAAC
**KRAS, exon 2 (codon 12 and 13**	GTGTGACATGTTCTAATATAGTCA	CTGTATCAAAGAATGGTCCTGCAC
**BRAF, exon 15**	TCATAATGCTTGCTCTGATAGG	GGCCAAAAATTTAATCAGTGG

For *EGFR*, DNA was amplified in a total volume of 30 μl containing 1.5 mM MgCl_2_, 1.25 U of Ampli Taq Gold ® DNA, 3 μl of GeneAmp 10x PCR buffer II (ABI Cat N808-0241), 200 μM of each dNTP (dATP, dCTP, dGTP and dTTP; Promega, Cat U1410), 300 nM of each primer and 250 ng of DNA. DNA was first denatured at 95°C for 5 minutes, followed by 38 cycles of PCR including denaturation at 95°C for 45 s, annealing for 45 s at 60°C and extension for 45 s at 72°C. At the end of the last cycle, the mixture was incubated at 72°C for 10 minutes. As for *KRAS* and *BRAF*, each PCR reaction was performed in a 25 μl volume mixture containing 250 ng of genomic DNA, 2.5 μl of both forward and reverse primers (0.1 μmol each) and 12.5 μl of GoTaq^®^Hot Start Green Master Mix (Promega). PCR amplification was carried out using the following conditions: 1 cycle at 95°C (15 minutes); 35 cycles at 94°C (30 seconds), at 52°C for *KRAS* and 50°C for *BRAF*(30 seconds), and at 72°C (30 seconds), and a final extension step at 72°C (7 minutes). All PCR reactions were performed in G- storm thermal cycler (Somerton Biotechnology Centre, UK). PCR amplicons were then checked using Qiaxcel capillary electrophoresis, where negative controls (non-template sample) were used and blood lymphocyte DNA from a healthy individual was used as a positive control of the amplification.

### Sanger sequencing for *EGFR*, *KRAS* and *BRAF*

PCR products were then purified using the QIAquick PCR Purification Kit (Qiagen, Crawley UK) according to manufacturer's instructions. All purified PCR products were then sequenced in both orientations by Sanger sequencing using the manufacturers' recommendations. Sequencing reactions were run on 3500Xl Genetic analyser (life technologies), and then purified using Big DyeSAM Solution and Big DyeXterminator solution (life technologies), to eliminate the excess of labelled ddNTPs. The sequence alignments were done with the BioEdit Sequence Alignment Editor and analysed using SeqScape software 2.5 (Applied Biosystems). The DNA sequence of *EGFR* gene was obtained from GenBank (*EGFR*-001, transcript ID: ENST00000275493). We compared the *KRAS* sequence against (*KRAS*-001, transcript ID: ENST00000311936), and *BRAF* sequence against (*BRAF*-001, transcript ID: ENST00000288602) as in Gene Bank. Detected mutations/polymorphisms were confirmed by at least two independent PCR amplifications and repeated sequencing reactions.

### Chromogenic in-situ hybridization (CISH) for *EGFR*

*EGFR* gene amplification was investigated by CISH in a subset of 18 cases which showed the highest combined H score expression. We aimed to investigate cases with scores of 6, 5 and 4 with at least a score of 2 in each of cytoplasmic and nuclear compartments. Score 6 was not detected in any of the studied cases. Cases studied with CISH included the 2 cases of BOTs which showed a score of 5 and 16 cases with score 4 (14 BOTs and 2 LGSCs). CISH was performed as previously described [39]. CISH was performed on 3 μm- thick FFPE tumour sections using ZytoDot CISH Implementation Kit, ZytoVision GmbH, Germany. Briefly, Slides were deparaffinised in xylene and immersed in 100% ethanol followed by water. Slides were then immersed in pre-treatment solution at 98°C for 17 minutes, then immediately washed with distilled water for 3 washes, 2 minutes each. Enzymatic digestion was done by incubating the slides with pepsin (2-3 drops) for 6 minutes at room temperature. The slides were then washed, dehydrated and air dried. Application of 15μl of the ready to use probe, (ZytoDot SPEC *EGFR* Probe, ZytoVision GmbH, Germany) was followed by application of coverslips, sealing of the edges and denaturing at 95°C for 5 minutes and hybridization at 37°C overnight in SPOT-Light^®^CISH™ Hybridizer (Invitrogen, life technologies). On the next day, a stringent wash was performed using 5× standard saline citrate (SCC) at 75° C for 7 minutes, followed by washing twice for 2 minutes. Sections were blocked with 3% H_2_O_2_, diluted with methanol for 10 minutes and PBS wash was performed twice for 2 minutes. Non-specific binding was blocked by applying the blocking solution and by incubating for 10 minutes. After incubation with a Mouse anti-Dig antibody for 30 minutes at room temperature, the procedure was continued by incubation with Anti mouse HRP-polymer and substrate chromogenic solution (DAB) for 30 minutes and counterstained with haematoxylin for 5s. The tissues were dehydrated in ethanol and coverslipped in mounting solution.

Slides were examined by light microscopy. The CISH signals were seen as dark brown dots. We counted 60 nuclei with a high power (x40) objective. Also the average *EGFR* GCN per nucleus for each tissue section was calculated [40]. A GCN of six or more in the nucleus in more than 50% of tumour cells indicate the presence of amplification of the *EGFR* gene, while 3-5 gene copies in > 50% of studied cells were considered aneuploidy. CISH scoring was evaluated by three researchers (RS, ML and RN).

### Statistical analysis

Statistical analysis was performed using SPSS 18. Mann-Whitney Test was used for 2 group comparison. Results were considered significant when p value < 0.05.

## CONCLUSION

This is the first study to thoroughly investigate the gene status and protein expression of EGFR in BOTs. Our study showed that nuclear expression of EGFR in BOTs and LGSCs was significantly higher compared to HGSCs or benign tumours, which lends support to the molecular similarity between BOTs and LGSCs. While *EGFR* gene mutation and amplification was not detected, gene polymorphisms were identified, which may have potential clinical significance.
